# The intersection of aging, pain, and opioid use disorder: a retrospective chart review from an outpatient opioid treatment clinic

**DOI:** 10.3389/fpain.2025.1666006

**Published:** 2025-09-18

**Authors:** Bethea A. Kleykamp, Hannah Smith, Erin Lynch, Aaron Greenblatt, Eric Weintraub

**Affiliations:** Psychiatry Department, University of Maryland School of Medicine, Baltimore, MD, United States

**Keywords:** opioid use disorder, overdose, older adults, aging, pain

## Abstract

**Introduction:**

Older adults represent a growing proportion of individuals with opioid use disorder (OUD) and adults 55 + are significantly more likely to experience a fatal overdose. This exploratory pilot study examined age-related differences in health and treatment characteristics among patients in outpatient opioid treatment to assess whether older adults show distinct patterns compared to younger patients, providing insight into this growing population.

**Methods:**

This retrospective chart review analyzed data from 79 patients (ages 23–70) seeking care at a low-threshold outpatient opioid treatment clinic. Data were extracted from electronic health records and included demographics, substance use, diagnoses, current pain, depression, quality of life, and treatment characteristics. Associations between age and clinical variables were analyzed using correlational, logistic regression, and repeated-measures ANCOVA methods.

**Results:**

Older age was predictive of past pain-related diagnoses and older adults (55+) longer histories of illicit opioid use (mean = 30 years) and tobacco smoking (mean = 43 years) compared to younger adults. While polysubstance use was more common among younger patients, fentanyl use was high across all ages (∼65%). Older adults received higher methadone doses and remained in treatment longer. Despite greater chronic exposure to opioids, age was not significantly associated with depression or quality of life scores at intake.

**Conclusions:**

Findings from this pilot study reveal age-related patterns in substance use, pain history, and treatment engagement among patients with OUD. The data suggest that older adults may face unique risks related to cumulative opioid exposure, while also demonstrating potential protective factors such as treatment retention. Integrated, age-responsive approaches are urgently needed to address the complex needs of this growing population.

## 1 Introduction

Opioid-related overdose deaths in the United States (US) have led to over three million years of life lost and a reduction in life expectancy nationwide (1). Adults aged 55 and older face significantly higher risk of fatal overdose, especially Black/African-American older adults (2–4). At the same time, there has been an over 50% increase in the number of people aged 55 and over seeking treatment for opioid use disorder (OUD) nationally (3). The average age of individuals in opioid treatment programs is also rising, with those aged 50–59 now representing the largest segment of the treatment population (5). These alarming trends are occurring alongside the exponential growth of the older adult population which is expected to nearly double in coming decades (6). Despite the growing number of older adults living with addictive disorders, including OUD, it is estimated that only 25% or less of substance use treatment programs include age-tailored care for older adults (7, 8).

Older adults bring distinct health and life circumstances that heighten their vulnerability to both opioid use disorder (OUD) and overdose. Age-related physiological changes, including reduced renal and hepatic clearance, altered pharmacokinetics, and increased central nervous system sensitivity, can intensify the effects of opioids and complicate medication management (9, 10). In addition, multimorbidity and polypharmacy are highly prevalent in this population, increasing the likelihood of drug–drug interactions and adverse events (11–14). Factors such as social isolation and functional decline further exacerbate risk and may delay detection or engagement in care (15–18). These intersecting biological and social vulnerabilities not only elevate overdose risk but also complicate the clinical detection and management of OUD, highlighting the need for treatment models that account for aging-specific vulnerabilities.

Pain is a particularly relevant comorbidity in older adults, who have higher rates of chronic pain than younger adults, and in individuals with OUD, among whom pain is highly prevalent. A recent meta-analysis found that more than half of patients prescribed opioid agonist treatment (methadone or buprenorphine) for OUD had self-reported or diagnosed current pain (60%) and those with current chronic pain was 44% (19). Methadone and buprenorphine are also prescribed for the treatment of pain although they are primarily prescribed for the treatment of OUD (20, 21). Pain management in the context of OUD treatment is often suboptimal: two-thirds of patients reporting that their pain was not treated through their OUD program, and pain is a primary reason cited for returning to the use of illicit opioids like heroin and fentanyl (22).

Despite evidence that pain is associated with illicit opioid use, our understanding of it and other age-related risk factors associated with OUD is limited. A review of the literature found that less than 1% of research published in leading aging and addiction journals included study populations wholly comprised of individuals age 50 and older (23–25). Furthermore, research studies often employ imprecise age classifications, with some studies defining “older adults” as anyone above the age of 25 or 40 (26, 27). The limited body of research related to older age and opioid use could hinder efforts to treat OUD and concomitant pain in this population. Given the rapid aging of society and increasing number of older adults impacted by OUD and overdose, more data are needed to determine the unique characteristics of different age groups. This pilot, exploratory study accounts for these gaps through a retrospective chart review of patients seeking treatment at an outpatient opioid treatment clinic in Baltimore, Maryland, the city with the highest overdose rates in the US (28, 29). The aim of our study was to examine age-related differences in patient health and treatment characteristics, with a focus on direct comparisons between the youngest patients (<34 years) and the oldest patients (55+), given the elevated risk of fatal overdose in older adults (2). We predicted that older adults would have a greater number of comorbid health disorders, including pain-related conditions, and that substance use patterns would be similar between age groups.

## 2 Methods

### 2.1 Participants and data

Data for this retrospective chart review were retrieved from electronic health records (EPIC and Methasoft) for all patients at the University of Maryland Addiction Treatment Center (UMATC) that completed an intake appointment from January 1 to June 30, 2023. UMATC is an outpatient, medication-based treatment clinic accepting self- and provider-referrals, including walk-ins. Intakes were conducted by counselors and prescribers. The clinic employs a low-threshold maintenance approach, focusing on harm reduction over abstinence with a focus on minimizing barriers to care. All procedures were approved by the University of Maryland School of Medicine Institutional Review Board ((Protocol Number: HP-00107138).

Data were collected as part of the clinic's standard intake process, which involves separate assessments by counselors and medical staff, and are initiated the day the patient seeks treatment. As represented in the Supplementary Data Sheet S1 (data dictionary), counselors complete a structured clinical intake including a comprehensive psychosocial assessment and standardized questionnaires (e.g., PHQ-9). Medical personnel conduct a physical exam and review of systems, problem and medication lists, and a toxicology screen (completed at intake). Substance use, including alcohol, is assessed broadly by medical staff during the physical exam, with patients asked about current use (type, frequency, amount). Current pain is assessed as part of the medical exam. All data are entered directly into the electronic health record (EPIC).

UMATC conducted 86 intake interviews over six months; seven cases were excluded due to substantial missing data including demographics (mean age of excluded cases = 44.9 years, range = 33−59). De-identified data were entered into REDCap by three reviewers (BK, HS, and EL). Following trial extractions, the team developed a data dictionary to ensure consistent definitions and coding across variables (see Supplementary Data Sheet S1). All data were extracted according to this dictionary and reviewed in duplicate in RedCap or Excel to minimize errors. Any discrepancies were first reconciled by BK and, when needed, resolved through discussion with HS and EL. Data included demographics, assessments for depression (Patient Health Questionnaire; PHQ) (30) and the Quality of Life Enjoyment and Satisfaction Questionnaire (Q-LES-Q) (31), self-reported substance use (opioids, tobacco, and alcohol), diagnoses (at baseline and past diagnoses in patient chart), and pain (i.e., past pain-related diagnoses at intake and past diagnoses, as well as self-report of current pain at intake). We did not apply a validated comorbidity index such as the Charlson Comorbidity Index to patient diagnoses because intake data lacked sufficient detail to reliably distinguish disease severity; instead, we report the prevalence of specific physical and psychiatric diagnoses. In addition, urine toxicology results were examined at intake (2023) and follow-up (July 2024) for the following substances: methadone, amphetamine, methamphetamine, cocaine, opiates, heroin, fentanyl, oxycodone, barbiturate, benzodiazepine, and buprenorphine. Time in treatment was also measured and calculated as date of initial screening and last date that medication for OUD was administered closest to follow-up.

### 2.2 Statistical analyses

All analyses were conducted using SPSS (v28). Descriptive statistics were used to characterize the sample and normality of variables was assessed visually. When normality was not present, non-parametric tests were utilized. Chi-square tests or analysis of variances were used to compare differences based on age group (young, middle-aged older adults) for demographic variables. In addition, associations between age and continuous variables were tested using Pearson's correlation (Spearman's rank-order correlations completed when Pearson's assumptions not met), while binary logistic regressions were conducted to test whether age predicted dichotomous outcomes. To examine change in urine toxicology results over time, and whether age moderated this change, a repeated-measures ANCOVA was used with time (baseline vs. follow-up) as the within-subjects factor and age as a covariate. All statistical tests were two-tailed with significance set at *p* < .05. Where relevant, confidence intervals, effect sizes, and sample sizes for specific analyses are reported in the results. Percents are reported out of total final sample size are reported in text unless otherwise noted.

## 3 Results

### 3.1 Demographics

As represented in Table 1, the final sample included 79 patients with ages ranging from 23 to 70 years (Mean/M = 44.66, Standard Deviation/SD = 12.23) with a relatively equal number of younger and older adults (young *n* = 22 ages 23–35; older *n* = 23 ages 55 to 70). Most patients identified as White (51.9%) or Black (44.3%) and were male (61.8%). The only variable that was significantly different by age was race [Table 1; Pearson *χ*²(4) = 10.32, *p* = .035]. Pairwise analyses indicated that there was a significant difference between younger and older age groups after Bonferroni correction with a greater number of older adults who identified as Black, and greater number of younger adults who identified as White [*χ*²(2) = 10.24, *p* = .006; adjusted *α*=.017]. There were no significant differences in sex distribution between the middle-aged group and other groups.

**Table 1:** Patient demographics.

Variable	Total (*N* = 79)	Young (*n* = 23)	Middle (*n* = 34)	Older (*n* = 22)	Chi-square	*p*-value
Age (years)	44.7 (12.2)	30.7 (3.2)	43.9 (5.5)	60.5 (4.5)		
Gender/sex^a^						
Female	26	10	9	7	2.05	0.359
Male	42	12	22	8		
Not specified	11	1	3	7		
Race						
Black/AA	35	5	15	15	10.32^a^	0.035
White/Caucasian	41	17	18	6		
Other	3	1	1	1		
Ethnicity						
Hispanic	5	2	2	1	0.37	0.831
Non-Hispanic	72	20	32	20		
Not specified	2	1	0	1		
Employment						
Unemployed	59	18	25	16	5.29	0.259
Employed	13	4	6	3		
Retired	2	0	0	2		
Not specified	5	1	3	1		
Education level						
<High school	20	3	12	5	8.98	0.175
High school/GED	31	14	9	8		
Some College/bachelors	20	5	7	7		
Not specified	8	1	2	2		
Medication type						
Buprenorphine	17	5	9	3	1.30	0.521
Methadone	62	18	25	19		

^a^ Significant difference between age group. Age groups include younger (≤35 years), middle-aged (36–54 years), and older adults (≥55).

### 3.2 Depression, and quality of life

In addition, at intake, patients reported mild depression on the PHQ (M = 5.9, SD = 6.8) and moderate life satisfaction on the Q-LES-Q (M = 54.6%, SD = 20). Spearman's rank-order correlations showed no significant association between age and depression/quality of life scores (ps > .05).

### 3.3 Self-reported opioid use

Most patients (70.3%) reported treatment for their opioid use in the last two years (45 of 64 patients who responded to this prompt; 70.3%), and age was not a significant predictor of treatment status [*B* = 0.036, SE = 0.024, Wald *χ*²(1) = 2.26, *p* = .133]. A shown in Table 2, the mean age for initiating non-medical opioid use was 24.3 years (SD = 10.5) and did not differ by age, but mean duration of use was greater for older adults (Mean = 30.0, SD = 15.6). Only four patients had a recorded history of opioid overdose in the charts reviewed, and most were age 55 or over (i.e., ages 25, 55, 65, and 70 years).

**Table 2:** Opioid Use and opioid Use disorder (OUD) treatment characteristics.

Variable	Total sample (*N* = 79)	Young (*n* = 23)	Middle (*n* = 34)	Older (*n* = 22)
Age of initiation of illicit opioid use	24.3 (10.5)	22.0 (5.0)	22.0 (8.9)	30.9 (14.9)
Duration of illicit opioid use (years)	26.9 (12.7)	8.5 (4.9)	21.9 (10.3)	30.0 (15.6)
Starting dose of OUD medication				
*Methadone (mg)*	46.05 (29.9)	33.0 (9.2)	47.9 (32.0)	63.89 (39.2)
*Buprenorphine (mg)*	9.17 (4.9)	7.00 (3.8)	8.4 (5.0)	13.33 (4.6)
Number remaining in treatment	10	0		5
Duration in treatment (days)	143.6 (175.0)	112.4 (127.7)	128.3 (185.4)	199.2 (195.4)

Data are means (SD) or counts. Age groups include younger (≤35 years), middle-aged (36–54 years), and older adults (≥55).

### 3.4 Self-reported tobacco and alcohol use

Nearly all (96%) of patients used smoked tobacco regularly at some point in their life (current = 65; former = 11) with a mean age of initiation of 16.3 years (SD = 5.2). Of the 65 current smokers (82.2% of the full sample), 62 smoked daily for an average of 12.0 cigarettes per day (SD = 8.7). Although age was not associated with age of initiation or cigarettes per day, it was significantly related to duration of smoking (Spearman's *ρ* = .91, *p* < .001). On average, the number of years smoking was 15.4 for young adults (≤35 years), 27.8 for middle-aged adults (36−54 years), and 43.3 for older adults (55 + years). Only a few patients of the total sample (*n* = 23) were asked about smoking cessation at intake, and most were not interested in quitting (*n* = 17; 73.9%). In addition to tobacco, patients self-reported their alcohol use (binary outcome yes/no). Thirteen (16.5%) patients of the total sample reported current alcohol use and age was a significant predictor with alcohol use more common as age increased [B = 0.057, SE = 0.027, Wald *χ*²(1) = 4.56, *p* = .033].

### 3.5 Urine toxicology

Toxicology results (positive yes/no) at intake and follow-up were available for 73 patients (6 missing). At intake, patients were positive for an average of 3.8 substances (SD = 1.5) with the most common being fentanyl (69.9%) and cocaine (52.1%). At follow-up, patients were positive for an average of 2.9 substances (SD = 1.5), with most common being fentanyl (64.4%) and cocaine (52.1%), as well as methadone (61.6%). The least common positive results (<10%) at any time point included heroin, barbiturates, amphetamine, methamphetamine, and oxycodone.

A repeated-measures ANCOVA was conducted to examine whether age was associated with change in the number of substances testing positive from baseline to follow-up. The within-subjects effect of time was not significant, F(1,71) = 0.31, *p* = .577, and the interaction between time and age was also not significant, F(1,71) = 1.34, *p* = .250, suggesting that substance use did not significantly change over time in treatment and age was not associated with change. However, descriptively participants tested positive for nearly one fewer substance at follow-up [mean difference = 0.99, 95% CI (0.68, 1.29)]. In addition, a significant between-subjects effect was observed for age, F(1,71) = 10.79, *p* = .002, indicating that younger patients tested positive for a greater number of substances across time points compared to older.

### 3.6 Diagnoses

Across the full sample, diagnostic codes utilized at the time of the intake appointment focused primarily on substance use disorders in addition to OUD, including cocaine use disorder (*n* = 45, 57%), cannabis use disorder (*n* = 16, 20.3%), and tobacco use disorder (*n* = 13, 16.5%). In addition, diagnostic codes related to testing for infectious and chronic disease were common (*n* = 65, 82.3%; i.e., sexual transmitted, tuberculosis, diabetes, lipid disorders, hypothyroidism). Overall, number of diagnoses per person ranged from 0 to 12 (M = 6.96, SD = 2.97) and age was not significantly associated with the number of visit diagnoses (Spearman's *ρ* = –.18, *p* = .062, *n* = 79). None of the diagnoses at intake were related to pain.

We also extracted data for previous diagnoses in the patient's chart (i.e., past diagnoses) and, of these, the number of diagnoses per person ranged from 0 to 10 (M = 5.35, SD = 3.66) and was not significantly related to patient age (Spearman's *ρ* = .19, *p* = .101, *n* = 79). The most frequent diagnostic categories for conditions listed included cardiovascular disease (*n* = 31; 39.2%), skin injuries/infections (*n* = 17; 21.5%), pulmonary/respiratory (*n* = 17; 21.5%), and infectious disease such as hepatitis C and HIV (*n* = 17; 21.5%). Type of comorbidity did not differ significantly between age groups (ps > 0.05).

### 3.7 Pain

As noted and shown in Table 3, no patients were diagnosed with pain-related conditions at intake. When reviewing past diagnoses, we found that 29 (36.7%) patients had a diagnosis that was related to pain, which we categorized into distinct groups for clarity. Among the 29 participants with pain-related diagnoses, the most common diagnostic category was cellulitis and other soft tissue infections (9 cases), followed by joint pain, arthritis, and gout (7), radicular and neuropathic pain (5), chest pain/angina (4), and focal extremity pain (4). Other less common pain conditions included headache (2), abdominal or epigastric pain (2), low back or generalized musculoskeletal pain (2), rhabdomyolysis (2), cervical pain (1), and peripheral vascular disease (1). The frequency of pain-related diagnoses per individual participant was one (*n* = 13), two (*n* = 11), three (*n* = 4), or five (*n* = 1). In addition to past diagnoses, 60 of the 79 patients (75.95%) were asked at intake whether they were currently experiencing any pain and approximately half responded “yes” (31 of 60; 51.67%) (Table 3). Binary logistic regressions were conducted to examine whether age predicted the likelihood of reporting pain at the time of intake or having a pain-related diagnosis in the past. For current pain at intake, age was not a significant predictor in logistic regression analyes (B = 0.27, Wald = 1.76, *p* = .185), but it was a significant predicter of past pain-related diagnosis (B = 0.49, Wald = 5.71, *p* = .017). This equated to an odds ratio of 1.63, indicating that for each additional year of age, the odds of have a past pain-related diagnosis increased by approximately 63%.

**Table 3:** Pain-related outcomes.

Variable	Total sample (*N* = 79)	Young (*n* = 23)	Middle (*n* = 34)	Older (*n* = 22)
Pain diagnosis at intake	0	0	0	0
Past pain diagnosis	29	7	9	13
Reporting pain at intake	31	8	12	11

Data are counts. Sample size for reporting pain at the time of treatment was 60 patients (data were missing for 19 patients). Age groups include younger (≤35 years), middle-aged (36–54 years), and older adults (≥55).

### 3.8 Treatment characteristics

After intake, most patients were treated with methadone (62; 78.5%) and a smaller portion with buprenorphine (17; 21.5%) with no difference in medication type by age (*p* > 0.05; Table 1). As shown in Table 2, the average starting dose was 46.1 mg (SD = 29.9) for methadone and 9.2 mg (4.9) for buprenorphine. Age was not related to buprenorphine dose (*p* > 0.05), but there was a significant positive correlation between age and starting methadone dose indicating that older patients tended to receive higher initial doses of methadone (r = .37, *p* = .014, *n* = 43). Mean starting doses of methadone were 33 mg for younger (≤35 years), 47.9 mg for middle age (36−54), and 63.9 mg for older adults (55+). Number of days in treatment, measured as time until discharge at follow-up, was a mean of 154.9 days (SD = 168.2) and older age was associated with longer time in treatment (r = .26, *p* = .021, *n* = 78; older cohort = 199.2 days vs. younger cohort = 112.4 days) (Table 2). The number of patients still in treatment at final follow-up was low overall (*n* = 10; 12.7%), but more common for the older adult cohort compared to younger (5 vs. 0 patients).

## 4 Discussion

This retrospective chart review of a university-affiliated outpatient opioid treatment clinic identified some age-related differences in health outcomes. Although age was not significantly associated with self-report of pain at intake, older adults were significantly more likely to have past pain-related diagnoses. Specifically, each additional year of age was associated with a 63% increase in the odds of having a pain-related diagnosis, underscoring the importance of addressing pain in the aging population with OUD. At the same time, age was not associated with the overall number of visit diagnoses, which may reflect the uniformly high comorbidity burden across this population, particularly in the context of fentanyl use, such that age-related differences in multimorbidity were less evident at treatment entry. Notably, pain-related diagnoses were absent from intake problem lists, suggesting that although providers routinely ask about pain at intake, it may not be prioritized by patients or providers in this setting, where immediate clinical focus is often placed on initiating medications for OUD (e.g., methadone, buprenorphine) and reducing illicit opioid use and near-term harms (e.g., infectious disease, overdose). At the same time, prior studies indicate that pain frequently shapes patterns of substance use and relapse risk, highlighting the importance of incorporating structured pain assessment and management into the early stages of OUD treatment to support both patient engagement and long-term outcomes (19, 22, 32).

The study found that a substantial majority of patients (82%) were currently smoking tobacco which is consistent with national data showing that tobacco use among individuals with OUD is both persistently high and nearly five times more prevalent than in the general population (33). Tobacco smoking promotes systemic low-grade inflammation, which explains, in part, its association with a range of pain-related conditions including chronic musculoskeletal pain, low back pain, and headaches (34–37). Notably, our study identified significantly greater lifetime tobacco exposure among older adults, with a mean smoking duration of 43 years which highlights the potential cumulative impact of tobacco use on pain-related morbidity in this population. Despite this, both our findings and previous research suggest that many patients in OUD treatment have limited interest in smoking cessation, underscoring the urgent need for innovative, tailored strategies to address tobacco use and its associated disease states within addiction treatment settings (38–40).

In contrast to tobacco use, which did not differ by age, our urinalysis results revealed a higher likelihood of polysubstance use among younger adults which can increase the risk of overdose and negatively impact OUD treatment outcomes (41). Although less common among older adults, polysubstance use was still present and remains clinically significant given age-related declines in metabolic clearance, which may heighten the risk of drug accumulation and overdose. Furthermore, fentanyl, a potent synthetic opioid and major contributor to overdose deaths, was detected in approximately two-thirds of patients at both intake and follow-up, with no differences observed by age. In addition, older adults in the sample reported an average of 30 years of illicit opioid use, compared to 8.5 years among younger adults (Table 2), reflecting more prolonged exposure. Chronic opioid use is associated with opioid-induced hyperalgesia (OIH), a condition in which long-term opioid exposure leads to increased pain sensitivity (42). Evidence also suggests that acute exposure to high-potency opioids like fentanyl can trigger hyperalgesia, even in the absence of chronic use (42, 43). Taken together, these findings highlight the co-occurrence of pain and opioid use, particularly in the context of fentanyl exposure, and highlight the importance of addressing pain management in OUD treatment across the lifespan.

Treatment-related findings further underscore age-related differences in opioid use and treatment trajectories. Older adults in the sample were prescribed higher methadone doses at treatment initiation and remained in care longer than younger patients. These differences may reflect greater treatment engagement, provider perceptions of stability or risk, or age-related physiological changes that affect opioid metabolism. However, the low absolute number of participants retained in treatment overall (*n* = 10) underscores the need for caution in interpretation and highlights the importance of developing strategies to support long-term treatment retention across age groups. Older adults were also more likely to test positive for methadone at intake, which could indicate prior enrollment in another treatment program or possible illicit methadone use to manage OUD or pain—though our study was not designed to examine these pathways. Lastly, despite older adults reporting more chronic and extensive opioid use histories, age was not significantly associated with depression symptoms or quality of life ratings at intake. This was unexpected, given the well-documented negative effects of long-term substance use on both mental health and quality of life. These findings may reflect the heterogeneity of the sample or the influence of other unmeasured factors, such as social support or resilience, that could buffer the impact of long-term opioid use in older adults (44).

### 4.1 Clinical implications

The findings from this pilot study have direct implications for practitioners caring for aging adults with pain and opioid use disorder. Our data show that older adults may present with long-standing opioid use histories, high rates of lifetime tobacco exposure, and untreated or under-documented pain condition. These characteristics may increase vulnerability to opioid-induced hyperalgesia, functional impairment, and poor quality of life. However, our study and others suggest that older adults may demonstrate higher levels of treatment engagement and retention, offering an important window for OUD- and pain-related interventions (45, 46). The American Society of Addiction Medicine (ASAM) has recommended that methadone and buprenorphine medications be considered for patients with both OUD and pain (47). For acute and chronic pain, dosing adjustments including increasing the dose or splitting it throughout the day may improve algesia (e.g., 3−4 smaller doses of methadone rather than 1 per day). Buprenorphine may be especially appropriate for older adults given its ceiling effect on respiratory depression and lower overdose risk. However, for some older patients, methadone might be their preferred treatment modality highlighting the importance of shared decision-making around pain management. Given the high prevalence of pain in this population and its influence on treatment trajectories, systematically addressing pain alongside OUD care is essential to optimizing engagement, reducing relapse risk, and improving long-term outcomes. As such, ASAM guidelines emphasize the importance of coordinated, multidisciplinary care involving addiction treatment providers, primary care clinicians, and pain specialists to ensure safe, effective, and individualized treatment.

### 4.2 Limitations

The generalizability of the analyses presented here are reduced by its retrospective design, as and inherent limitations associated with conducting research with tools that are created for clinical purposes (i.e., medical charts). Thus, because the study was observational and cross-sectional for many variables, causality cannot be inferred. In addition, as a cross-sectional pilot study relying on a convenience sample, the analyses were not sufficiently powered to detect small to moderate between-group differences, thereby limiting the ability to adjust for potential confounders and covariates in statistical models. Given the number of comparisons conducted, the risk of Type I error is also elevated, and findings should therefore be interpreted with caution. Generalizability of findings is also limited because some variables (e.g., overdose history, pain at intake) were based on incomplete clinical documentation or patient self-report, and no participants over age 70 were included, restricting insight into this growing age group. Despite these limitations and given the significant lack of research focused on older adults in addiction treatment, the findings presented are instrumental in shedding light on older adults who are an understudied and high-risk population (23–25).

## 5 Conclusions

This study contributes to a growing body of evidence highlighting the complex and understudied intersection of aging, pain, and opioid use disorder. Our findings suggest that while older adults in treatment may present with lower rates of polysubstance use and greater engagement with care, they also report more chronic and prolonged opioid exposure and are more likely to have a history of pain-related diagnoses. These factors may elevate their vulnerability to conditions such as opioid-induced hyperalgesia and complicate efforts to manage pain safely and effectively. Both pain and polysubstance use have emerged as risk factors for return to substance use and overdose (11, 48–51). In addition, the high prevalence of long-term tobacco use, particularly among older adults, raises concerns about compounding inflammation and pain burden over time. Taken together, the findings underscore the need for integrated, age-informed models of addiction care that address substance use and pain later life (52). Future research should build on these findings to identify modifiable factors that promote health and treatment retention among older adults living with OUD.

## Data Availability

The raw data supporting the conclusions of this article will be made available by the authors, without undue reservation.

## References

[1] Hébert AH Hill AL. Impact of opioid overdoses on US life expectancy and years of life lost, by demographic group and stimulant co-involvement: a mortality data analysis from 2019 to 2022. Lancet Reg Health Am. (2024) 36:1–11. 10.1016/j.lana.2024.100813 PMC11228948 38978785

[2] Jones A Santos-Lozada A Perez-Brumer A Latkin C Shoptaw S El-Bassel N. Age-specific disparities in fatal drug overdoses highest among older black adults and American Indian/Alaska native individuals of all ages in the United States, 2015−2020. Int J Drug Policy. (2023) 114:103977. 10.1016/j.drugpo.2023.103977 36863284 PMC10050114

[3] Mason M Soliman R Kim HS Post LA. Disparities by sex and race and ethnicity in death rates due to opioid overdose among adults 55 years or older, 1999 to 2019. JAMA Netw Open. (2022) 5(1):e2142982. 10.1001/jamanetworkopen.2021.42982 35015062 PMC8753495

[4] Harris RA. Drug overdose deaths among non-hispanic black men in the U.S.: age-specific projections through 2025. AJPM Focus. (2023) 2(1):1–7. 10.1016/j.focus.2022.100063 PMC10299749 37377540

[5] Han B Polydorou S Ferris R Blaum CS Ross S McNeely J. Demographic trends of adults in New York city opioid treatment programs–an aging population. Subst Use Misuse. (2015) 50(13):1660–7. 10.3109/10826084.2015.1027929 26584180

[6] United Nations Department of Economic and Social Affairs. World Population Ageing 2019. Statistical Papers, Series A: Population and Vital Statistics Report. New York, NY: United Nations (2020). p. 60–00. 10.18356/6a8968ef-en

[7] Substance Abuse and Mental Health Services Administration. Treating Substance Use Disorder in Older Adults. Treatment Improvement Protocol (TIP) Series 26. Rockville, MD: Substance Abuse and Mental Health Services Administration (2020).

[8] Huhn AS Strain EC Tompkins DA Dunn KE. A hidden aspect of the US opioid crisis: rise in first-time treatment admissions for older adults with opioid use disorder. Drug Alcohol Depend. (2018) 193:142–7. 10.1016/j.drugalcdep.2018.10.002 30384321 PMC6242338

[9] Carew AM Comiskey C. Treatment for opioid use and outcomes in older adults: a systematic literature review. Drug Alcohol Depend. (2018) 182:48–57. 10.1016/j.drugalcdep.2017.10.007 29136566

[10] Gronich N. Central nervous system medications: pharmacokinetic and pharmacodynamic considerations for older adults. Drugs Aging. (2024) 41(6):507–19. 10.1007/s40266-024-01117-w 38814377 PMC11193826

[11] Han BH Tuazon E Wei MY Paone D. Multimorbidity and inpatient utilization among older adults with opioid use disorder in New York city. J Gen Intern Med. (2022) 37(7):1634–40. 10.1007/s11606-021-07130-8 34643872 PMC9130354

[12] Han BH. Aging, multimorbidity, and substance use disorders: the growing case for integrating the principles of geriatric care and harm reduction. Int J Drug Policy. (2018) 58:135–6. 10.1016/j.drugpo.2018.06.005 29957564 PMC6112977

[13] Salive ME. Multimorbidity in older adults. Epidemiol Rev. (2013) 35(1):75–83. 10.1093/epirev/mxs009 23372025

[14] Hoel RW Giddings Connolly RM Takahashi PY. Polypharmacy management in older patients. Mayo Clin Proc. (2021) 96(1):242–56. 10.1016/j.mayocp.2020.06.012 33413822

[15] Han BH Cotton BP Polydorou S Sherman SE Ferris R Arcila-Mesa M Geriatric conditions among middle-aged and older adults on methadone maintenance treatment: a pilot study. J Addict Med. (2022) 16(1):110–3. 10.1097/ADM.0000000000000808 33395146 PMC8243387

[16] Yang T-C Shoff C Kim S Shaw BA. County social isolation and opioid use disorder among older adults: a longitudinal analysis of medicare data, 2013–2018. Soc Sci Med. (2022) 301:114971. 10.1016/j.socscimed.2022.114971 35430465 PMC10211474

[17] Frueh L Collins AB Newman R Barnett NP Rich JD Clark MA Multi-level influences on increased overdose risk behaviors during the COVID-19 pandemic among people who use drugs in Rhode Island: a qualitative investigation. Harm Reduct J. (2023) 20(1):14. 10.1186/s12954-023-00741-w 36739417 PMC9898862

[18] Dufort A Samaan Z. Problematic opioid use among older adults: epidemiology, adverse outcomes and treatment considerations. Drugs Aging. (2021) 38(12):1043–53. 10.1007/s40266-021-00893-z 34490542 PMC8421190

[19] Yang J Jung M Picco L Grist E Lloyd-Jones M Giummarra M Pain in people seeking and receiving opioid agonist treatment: a systematic review and meta-analysis of prevalence and correlates. Addiction. (2024) 119(11):1879–901. 10.1111/add.16574 38886901

[20] Spreen LA Dittmar EN Quirk KC Smith MA. Buprenorphine initiation strategies for opioid use disorder and pain management: a systematic review. Pharmacotherapy. (2022) 42(5):411–27. 10.1002/phar.2676 35302671 PMC9310825

[21] Hanna V Senderovich H. Methadone in pain management: a systematic review. J Pain. (2021) 22(3):233–45. 10.1016/j.jpain.2020.04.004 32599153

[22] Ellis MS Kasper Z Cicero T. Assessment of chronic pain management in the treatment of opioid use disorder: gaps in care and implications for treatment outcomes. J Pain. (2021) 22(4):432–9. 10.1016/j.jpain.2020.10.005 33197581

[23] Rosen D Engel RJ Hunsaker AE Engel Y Detlefsen EG Reynolds CF. Just say know: an examination of substance use disorders among older adults in gerontological and substance abuse journals. Soc Work Public Health. (2013) 28(3–4):377–87. 10.1080/19371918.2013.774668 23731426 PMC4047645

[24] Rosen D Engel RJ Beaugard C Davis N Cochran G. Baby boomer’s substance abuse and researcher indifference. J Gerontol Soc Work. (2019) 62(1):16–28. 10.1080/01634372.2018.1530715 30289373

[25] Kleykamp BA Kulak JA. Cigarette use among older adults: a forgotten population. Am J Public Health. (2023) 113(1):27–9. 10.2105/AJPH.2022.307151 36413703 PMC9755936

[26] Dingle T Bowen S. Evaluating substance use treatment efficacy for younger and older adults. Addict Behav. (2021) 112:106618. 10.1016/j.addbeh.2020.106618 32889444

[27] Sichel CE Winetsky D Campos S O'Grady MA Tross S Kim J Patterns and contexts of polysubstance use among young and older adults who are involved in the criminal legal system and use opioids: a mixed methods study. J Subst Abuse Treat. (2022) 143:108864. 10.1016/j.jsat.2022.108864 36242819 PMC11726775

[28] Maryland Department of Health. Overdose Data Portal: Fatal Overdose Historic Trends. Baltimore, MD: Maryland Department of Health (2024). Available online at: https://health.maryland.gov/dataoffice/Pages/mdh-dashboards.aspx#Overdose (Accessed September 7, 2025).

[29] Centers for Disease Control and Prevention. CDC WONDER Database. Atlanta, GA: US Department of Health and Human Services, Centers for Disease Control and Prevention (2024). Available online at: https://wonder.cdc.gov/ (Accessed September 7, 2025).

[30] Kroenke K Spitzer RL Williams JB. The PHQ-9: validity of a brief depression severity measure. J Gen Intern Med. (2001) 16(9):606–13. 10.1046/j.1525-1497.2001.016009606.x 11556941 PMC1495268

[31] Endicott J Nee J Harrison W Blumenthal R. Quality of life enjoyment and satisfaction questionnaire: a new measure. Psychopharmacol Bull. (1993) 29(2):321–6. 8290681

[32] Edwards KA Buonora MJ Merlin JS Liebschutz JM. Recent advances in the treatment of chronic pain and substance use disorders. Curr Opin Psychol. (2025) 62:101977. 10.1016/j.copsyc.2024.101977 39705790 PMC11867877

[33] Weinberger AH Gbedemah M Wall MM Hasin DS Zvolensky MJ Goodwin RD. Cigarette use is increasing among people with illicit substance use disorders in the United States, 2002−14: emerging disparities in vulnerable populations. Addiction. (2018) 113(4):719–28. 10.1111/add.14082 29265574 PMC6369915

[34] Shiri R Karppinen J Leino-Arjas P Solovieva S Viikari-Juntura E. The association between smoking and low back pain: a meta-analysis. Am J Med. (2010) 123(1):87.e7–87.e35. 10.1016/j.amjmed.2009.05.028 20102998

[35] Dai Y Huang J Hu Q Huang L Wu J Hu J. Association of cigarette smoking with risk of chronic musculoskeletal pain: a meta-analysis. Pain Physician. (2021) 24(8):495. 34793634

[36] Błaszczyk B Martynowicz H Przegrałek J Niemiec P Straburzyński M Budrewicz S Smoking in primary headaches – a systematic review and meta-analysis. J Headache Pain. (2025) 26(1):133. 10.1186/s10194-025-02076-2 40468190 PMC12139338

[37] LaRowe LR Ditre JW. Pain, nicotine, and tobacco smoking: current state of the science. Pain. (2020) 161(8):1688–93. 10.1097/j.pain.0000000000001874 32701828 PMC8924914

[38] Richter KP McCool RM Okuyemi KS Mayo MS Ahluwalia JS. Patients’ views on smoking cessation and tobacco harm reduction during drug treatment. Nicotine Tob Res. (2002) 4(Suppl_2):S175–S82. 10.1080/1462220021000032735 12573178

[39] Bjørnestad ED Vederhus J-K Clausen T. High smoking and low cessation rates among patients in treatment for opioid and other substance use disorders. BMC Psychiatry. (2022) 22(1):649. 10.1186/s12888-022-04283-6 36261791 PMC9583489

[40] Guydish J Kapiteni K Le T Campbell B Pinsker E Delucchi K. Tobacco use and tobacco services in California substance use treatment programs. Drug Alcohol Depend. (2020) 214:108173. 10.1016/j.drugalcdep.2020.108173 32693199 PMC7439769

[41] Gladden RM O'Donnell J Mattson CL Seth P. Changes in opioid-involved overdose deaths by opioid type and presence of benzodiazepines, cocaine, and methamphetamine - 25 states, July-December 2017 to January-June 2018. MMWR Morb Mortal Wkly Rep. (2019) 68(34):737–44. 10.15585/mmwr.mm6834a2 31465320 PMC6715260

[42] Lee M Silverman SM Hansen H Patel VB Manchikanti L. A comprehensive review of opioid-induced hyperalgesia. Pain Physician. (2011) 14(2):145. 10.36076/ppj.2011/14/145 21412369

[43] Mauermann E Filitz J Dolder P Rentsch KM Bandschapp O Ruppen W. Does fentanyl lead to opioid-induced hyperalgesia in healthy volunteers?: a double-blind, randomized, crossover trial. Anesthesiology. (2016) 124(2):453–63. 10.1097/ALN.0000000000000976 26655493

[44] Yang C Xia M Han M Liang Y. Social support and resilience as mediators between stress and life satisfaction among people with substance use disorder in China. Front Psychiatry. (2018) 9:436. 10.3389/fpsyt.2018.00436 30386257 PMC6198788

[45] Francis AG Croll J Vaishnav H Langdon K Beaudoin FL. Treatment retention in older versus younger adults with opioid use disorder: a retrospective cohort analysis from a large single center treatment program. R I Med J (2013). (2021) 104(1):51–4. 33517601

[46] Weinstein ZM Kim HW Cheng DM Quinn E Hui D Labelle CT Long-term retention in office based opioid treatment with buprenorphine. J Subst Abuse Treat. (2017) 74:65–70. 10.1016/j.jsat.2016.12.010 28132702 PMC5312773

[47] Cunningham C Edlund MJ Fishman M Gordon AJ Jones HE Langleben D The ASAM national practice guideline for the treatment of opioid use disorder: 2020 focused update. J Addict Med. (2020) 14(2S Suppl 1):1–91. 10.1097/ADM.0000000000000633 32511106

[48] Hoots B Vivolo-Kantor A Seth P. The rise in non-fatal and fatal overdoses involving stimulants with and without opioids in the United States. Addiction. (2020) 115(5):946–58. 10.1111/add.14878 31912625 PMC11301974

[49] Palis H Gan W Xavier C Desai R Scow M Sedgemore K-o Association of opioid and stimulant use disorder diagnoses with fatal and nonfatal overdose among people with a history of incarceration. JAMA Network Open. (2022) 5(11):e2243653. 10.1001/jamanetworkopen.2022.43653 36416821 PMC9685494

[50] Cano M Oh S Salas-Wright CP Vaughn MG. Cocaine use and overdose mortality in the United States: evidence from two national data sources, 2002–2018. Drug Alcohol Depend. (2020) 214:108148. 10.1016/j.drugalcdep.2020.108148 32702620 PMC7423708

[51] Tori ME Larochelle MR Naimi TS. Alcohol or benzodiazepine co-involvement with opioid overdose deaths in the United States, 1999−2017. JAMA Netw Open. (2020) 3(4):e202361. 10.1001/jamanetworkopen.2020.2361 32271389 PMC7146101

[52] Jones KF Beiting KJ Ari M Lau-Ng R Landi AJ Kelly L Age-friendly care for older adults with substance use disorder. Lancet Healthy Longev. (2023) 4(10):e531–e2. 10.1016/S2666-7568(23)00174-5 37804842 PMC12718471

