# Supplementary material for: The intersection of aging, pain, and opioid use disorder: a retrospective chart review from an outpatient opioid treatment clinic
Source: Front Pain Res (Lausanne). 2025 Sep 18;6:1666006. doi: 10.3389/fpain.2025.1666006 (PMC12488411; doi:10.3389/fpain.2025.1666006)
Supplement: Supplementary file 1 [file Datasheet1.pdf]

**Provider/Medical Screening (Epic)**		
Items	Response/Response Type:	Location:
Was the provider screening conducted?	Yes;<br>No	
Completion Date	Text (date)	Date form was completed by reviewer
Date of Provider Screening	Text (date)	Date that intake screening was completed by provider
Provider	Text (name) (ex. Eric Weintraub)	Name of provider that conducted the screening
Attending Provider	Text (name)	Name of the provider that supervised intake screening
Reviewer Initials	Text	Name of reviewer
**Patient Demographics**		
Date of birth	Text (date)	See left Margin of chart
Age (years)	Calculated field	Will be automatically generated based on date of birth
Race	White; Black or African American; American Indian or Alaskan Native; Native Hawaiian or Pacific Islander; Asian; Other	History at bottom of office visit chart → Social Identity
Ethnicity	Hispanic or Latino; NOT Hispanic or Latino;	History at bottom of office visit chart → Social Identity
**Socioeconomic Status**		
Housing Status	Stable; Unstable	Social History in EPIC – Lives in
Employment status	Employed; Unemployed; Unreported	Social History in EPIC – Occupation/income
Educational status	Less than high school;<br>High school graduate;<br>GED;<br>Some college;<br>Associates degree;<br>Bachelor's degree;<br>Graduate or professional degree	Social History in EPIC – Highest level of education
**Tobacco/Nicotine**		
Does the patient currently use any tobacco/nicotine products?	Yes; No;	Substance Use History chart
Types of tobacco/nicotine use	Cigarettes;<br>Smokeless;<br>E-cigarettes;	Substance Use History chart – Route

	Cigars/Cigarillos/Little Cigars;<br>Pipes;<br>Waterpipe/Hookah;<br>Snus;<br>Other;	
If smoking, how many cigarettes?	Open-ended	Substance Use History chart – Amount
What is the patient's frequency of cigarette use?	Open-ended	Substance Use History chart – Frequency
When did the patient start using tobacco? (Age in years)	Text	Substance Use History chart – Age of Onset
Is smoking cessation addressed (e.g., in treatment plan or elsewhere)?	Yes; No;	Assessment & Plan
If yes, please describe	Open-ended	
If yes, please describe	Open-ended	
Additional information related to tobacco or nicotine use?	Open-ended	Please list anything related to tobacco use that you see that could be relevant including treatment planning or general chart notes.
**Opioids**		
Does the patient currently use any opioids?	Yes; No;	Substance Use History chart
Which types of opioids has the patient used?	Heroin/fentanyl;<br>Prescription opioids;<br>Both;	
Route of Administration	IV;<br>Snort;<br>PO;<br>Smoke;<br>Other;	Substance Use History chart – Route
How much is the patient using?	Open-ended	
What is the frequency of opioid use?	Open-ended	
When did the patient start using recreational opioids? (Age in years)	Text	Substance Use History chart – Age of Onset
Has the patient received care from an OTP in the last 2 years?	Yes<br>No	Previous Treatment History
Treatment history (e.g., has the patient used evidence-	Yes<br>No	Previous Treatment History

based treatments before for opioid use)		
When did the patient last receive treatment for opioid use?	Open-ended	Previous Treatment History
How long was the patient last in treatment for opioid use?	Open-ended	Previous Treatment History
Is the patient taking prescribed opioids for pain?	Yes;<br>No;	Previous Treatment History
Treatment plan for opioid use	Methadone; Naltrexone; Buprenorphine or Buprenorphine/Nalaxone	Initiate Treatment With
If methadone, start dose		
If Buprenorphine or Buprenorphine/Naltrexone, start dose		
If Naltrexone, start dose		
If Other, please list name of medication and start dose		
Is opioid harm reduction addressed including Naloxone, needle exchange, etc?	Yes;<br>No;	Assessment & Plan – patient may be offered Naloxone and accept/decline OR Medication List will have Naloxone listed with the date of intake
If yes, please describe	Open-ended	
Additional information related to opioid use?	Open-ended	
**Alcohol**		
Does the patient currently use alcohol?	Yes;<br>No;	Substance Use History chart
Type of alcohol use	Beer;<br>Wine;<br>Liquor;<br>Other;	Substance Use History chart – Amount
If other, what other types of alcohol does the patient use?	Open-ended	
How much does the patient drink per sitting?	Open-ended	Substance Use History chart – Amount
When did the patient start using alcohol? (Age in years)	Text	Substance Use History chart – Age of Onset
What is the frequency of alcohol use?	Open-ended	Substance Use History chart – Frequency

Additional information related to alcohol use?	Open-ended	
**Cannabis**		
Does the patient currently use cannabis?	Yes;<br>No;	Epic (Substance Use History chart)
Type of cannabis use	Smoke;<br>Vaping;<br>Edibles;<br>Other;	Epic (Substance Use History chart – Route)
If other, what other types of cannabis does the patient use?	Open-ended	
How much is the patient using?	Open-ended	Substance Use History chart – Amount
What is the frequency of cannabis use?	Open-ended	Substance Use History chart – Frequency
When did the patient first start using cannabis? (Age in years)	Text	Substance Use History chart – Age of Onset
Additional information related to cannabis use?	Open-ended	
**Stimulants**		
Does the patient currently use stimulants?	Yes;<br>No;	Substance Use History chart
Is the patient currently using prescribed stimulants?	Yes;<br>No;	MD PDMP Dispense History
Type of stimulant use (e.g., cocaine, amphetamine, methamphetamine)	Cocaine;<br>Amphetamines;<br>Methamphetamines;<br>MDMA (Ecstasy/Molly);<br>Prescription stimulant misuse (eg. Adderall, Ritalin);<br>Other;	Substance Use History chart – Amount
If other, what other types of stimulants does the patient use?	Open-ended	
Route of administration	IV;<br>Snort;<br>PO;<br>Smoke;<br>Other;	Substance Use History chart – Route

How much is the patient using?	Open-ended	Substance Use History chart – Amount
What is the frequency of stimulant use?	Open-ended	Substance Use History chart – Frequency
When did the patient start using stimulants? (Age in years)	Text	Substance Use History chart – Age of Onset
Additional information related to stimulant use?	Open-ended	
**Benzodiazepines**		
Does the patient currently use benzodiazepines?	Yes;<br>No;	Substance Use History chart
Is the patient currently using prescribed benzodiazepines?	Yes;<br>No;	MD PDMP Dispense History
Type of benzodiazepine use	Diazepam (Valium);<br>Alprazolam (Xanax);<br>Lorzepam (Ativan);<br>Clonazepam (Klonopin);<br>Other;	
If other, what other types of benzodiazepines does the patient use?	Open-ended	
Route of administration	IV;<br>Snort;<br>PO;<br>Smoke;<br>Other;	Substance Use History chart – Route
How much is the patient using?	Open-ended	Epic (Substance Use History chart – Amount)
What is the frequency of benzodiazepine use?	Open-ended	Epic (Substance Use History chart – Frequency)
When did the patient start using benzodiazepines? (Age in years)	Text	Epic (Substance Use History chart – Age of Onset)
Additional information related to benzodiazepine use?	Open-ended	
**Other Illegal or Recreational Drugs**		
Does the patient currently use any other illegal or recreational drugs?	Yes;<br>No;	Epic (Substance Use History chart)
If yes, please describe	Open-ended	

**Pain Screening (yes/no)**		
**Physical Exam**		
Blood Pressure:		
Pulse:		
Weight:		
BMI:		
**Mental Status Exam**		
Musculoskeletal:		
**Referral to HARP Services**		
Primary Care:		
Infectious Disease Treatment:		
Psychiatric Treatment:		
**Tobacco Use**		
Tobacco frequency, amount, duration, pack years:		Find under Additional Documentation, Encounter Info: History and Tobacco Use
Smokeless Tobacco:		Find under Additional Documentation, Encounter Info: History and Tobacco Use.
Tobacco Cessation:		Find under Additional Documentation, Encounter Info: History and Tobacco Use.
Comments:		Find under Additional Documentation, Encounter Info: History and Tobacco Use.
**Vaping Use**		
Vaping:		Find under Additional Documentation, Encounter Info: History and Vaping Use.
**Key Terms**		
Does the patient's chart reference any of the following terms?	Precipitated withdrawal;<br>Xylazine;<br>Tranq;<br>Menthol;<br>Newport;<br>Tobacco harm reduction;<br>Mobility	Use Ctrl+F to find terms in text.
If () is referenced, please describe	Open-ended	
**Visit Diagnoses**		
Does the patient have any visit diagnoses listed?	Yes;<br>No	
How many visit diagnoses does the patient have?	1;<br>2;<br>3;<br>4;<br>5;	

	6;<br>7;<br>8;<br>9;<br>10;<br>10+;	
Visit Diagnosis #1 – #10	ICD-10 code	
(10+) Please list other visit diagnoses (ICD-10)	ICD-10 code	
**Problem List**<br>Note: Please limit problem list to the past 10 years from their visit.		
Does the patient have any diagnoses listed in their problem list?	Yes;<br>No	
How many diagnoses does the patient have in their problem list?	1;<br>2;<br>3;<br>4;<br>5;<br>6;<br>7;<br>8;<br>9;<br>10;<br>10+;	
Problem List #1 – #10	ICD-10 Code	
Date of Problem List Diagnosis #1:	Text	
(10+) Please list other diagnoses (ICD-10) and date of diagnosis.		Separate different ICD-10 codes with a semicolon (;) Ex. F32.A, M-D-Y;
**Medications**		
Does the patient take any medications besides their MOUD (methadone, buprenorphine etc.)?	Yes;<br>No	
How many medications does the patient take?	1;<br>2;<br>3;<br>4;<br>5;<br>6;<br>7;<br>8;<br>9;<br>10;<br>10+;	

Medication #1 – #10	Medication name and dosage	
For treatment of #1 – #10	Condition medication is prescribed for	
Last refill date for Medication #1 – #10	Text (date)	
(10+) Please list other medications with their doses, last refill date and what they treat:	Medication name, dosage and condition medication is prescribed for	Separate different medications with a semicolon (;) Ex. Prozac 30mg., M-D-Y, Depression;

**Laboratory Test Results (Methasoft, at intake)**		
Items	Response/Response Type:	Location:
Was the intake laboratory test collected?	Yes;<br>No	
Completion Date	Text (date)	Date form was completed by reviewer
Date of Provider Screening	Text (date)	Date that intake screening was completed by provider
Reviewer Initials	Text	Name of reviewer
Intake Clinic Urinalysis Result	Mtd (Methadone);<br>Mtab (Methadone Metabolites);<br>Amph (Amphetamines);<br>MAmph (Methamphetamines);<br>Coc (Cocaine);<br>Ops (Opiates);<br>Her (Heroin);<br>Fen (Fentanyl);<br>Oxy (Oxycodone);<br>Barb (Barbiturates);<br>Bzp (Benzodiazepines);<br>THC (Marijuana);<br>EtOH (Alcohol);<br>Prop (Propoxyphene);<br>Bup (Buprenorphine);<br>Negative	Methasoft (Under Drug Screen Results)
Observed?	Yes;<br>No;	

**Opioid Treatment Program Initial Screening Form (Epic)**		
Items	Response/Response Type:	Location:

Was the initial screening form completed?	Yes;<br>No	
Completion Date	Text (date)	Date form was completed by reviewer
Date of Counselor Screening	Text (date)	Date that intake screening was completed by counselor
Reviewer Initials	Text	Name of reviewer
Assigned Sex	Female; Male; Other;	Sex assigned at birth
Gender Identity	Female; Male; Other;	Gender identity
Type of insurance	Medicare; Medicaid; Private; Other;	Insurance
**Special Treatment Eligibility / Pertinent Legal Issues**		
Do any of the following circumstances apply to the patient?	Released from jail/prison in the last 6 months;<br>Awaiting court hearing;<br>Outstanding warrant;<br>Court-ordered treatment;<br>None	

**Initial Counselor Visit (Epic)**		
Items	Response/Response Type:	Location:
Was the intake counselor's assessment completed?	Yes;<br>No	
Completion Date	Text (date)	Date form was completed by reviewer
Date of Counselor Screening	Text (date)	Date that intake screening was completed by counselor
Reviewer Initials	Text	Name of reviewer
**Patient Health Questionnaire (PHQ-9)**		
Was this form completed?	Yes;<br>No	
Little interest or pleasure in doing things	Not at all;<br>Several days;<br>More than half the days;<br>Nearly every day	
Feeling down, depressed, or hopeless	Not at all;<br>Several days;<br>More than half the days;<br>Nearly every day	
Trouble falling or staying asleep, or sleeping too much	Not at all;<br>Several days;<br>More than half the days;<br>Nearly every day	
Feeling tired or having little energy	Not at all;<br>Several days;<br>More than half the days;	

	Nearly every day	
Poor appetite or overeating	Not at all;<br>Several days;<br>More than half the days;<br>Nearly every day	
Feeling bad about yourself -- or that you are a failure or have let yourself or your family down	Not at all;<br>Several days;<br>More than half the days;<br>Nearly every day	
Trouble concentrating on things, such as reading the newspaper or watching television	Not at all;<br>Several days;<br>More than half the days;<br>Nearly every day	
Moving or speaking so slowly that other people could have noticed? Or the opposite -- being so fidgety or restless that you have been moving around a lot more than usual?	Not at all;<br>Several days;<br>More than half the days;<br>Nearly every day	
Thoughts that you would be better off dead or of hurting yourself in some way	Not at all;<br>Several days;<br>More than half the days;<br>Nearly every day	
Total Score	Open-ended	
If you checked off any problems, how difficult have these problems made it for you to do your work, take care of things at home, or get along with other people?	Not applicable (0 PHQ-9 score);<br>Not difficult at all;<br>Somewhat difficult;<br>Very difficult;<br>Extremely difficult;	
**Addictions Assessment OP: Infectious Disease Assessment**		
Was this portion of the addictions assessment completed?	Yes;<br>No	
Last physical exam	Text (date)	
Do you have any infectious diseases?	Yes;<br>No;	
Have you received the Hepatitis A vaccine?	Yes;<br>No;	
Have you received the Hepatitis B vaccine?	Yes;<br>No;	
Have you ever seen a provider for a Hep C diagnosis PRIOR to treatment?	Yes;<br>No;	

Completed treatment?	Yes;<br>No;	
Have you been tested lately for HIV?	Yes;<br>No;	
When was the patient last tested for HIV?	Open-ended	
HIV Risk Factors	Open-ended	
Have you been tested lately for TB?	Yes;<br>No	
When was the patient last tested for TB?	Open-ended	
**Quality of Life Enjoyment and Satisfaction Questionnaire - Short Form**		
Was this form completed?	Yes;<br>No	
... physical health?	Very Poor;<br>Poor;<br>Fair;<br>Good;<br>Very Good	
... mood?	Very Poor;<br>Poor;<br>Fair;<br>Good;<br>Very Good	
... work?	Very Poor;<br>Poor;<br>Fair;<br>Good;<br>Very Good	
...household activities?	Very Poor;<br>Poor;<br>Fair;<br>Good;<br>Very Good	
...social relationships?	Very Poor;<br>Poor;<br>Fair;<br>Good;<br>Very Good	
...family relationships?	Very Poor;<br>Poor;<br>Fair;<br>Good;<br>Very Good	
...leisure time activities?	Very Poor;	

	Poor;<br>Fair;<br>Good;<br>Very Good	
...ability to function in daily life?	Very Poor;<br>Poor;<br>Fair;<br>Good;<br>Very Good	
...sexual drive, interest and/or performance?	Very Poor;<br>Poor;<br>Fair;<br>Good;<br>Very Good	
...economic status?	Very Poor;<br>Poor;<br>Fair;<br>Good;<br>Very Good	
...living/housing situation?	Very Poor;<br>Poor;<br>Fair;<br>Good;<br>Very Good	
... ability to get around physically without feeling dizzy or unsteady or falling?	Very Poor;<br>Poor;<br>Fair;<br>Good;<br>Very Good	
...your vision in terms of ability to do work or hobbies?	Very Poor;<br>Poor;<br>Fair;<br>Good;<br>Very Good	
...overall sense of well-being?	Very Poor;<br>Poor;<br>Fair;<br>Good;<br>Very Good	
… medication?	Very Poor;<br>Poor;<br>Fair;<br>Good;<br>Very Good	
Raw Total Score	Open-ended	
...How would you rate your overall life satisfaction and	Very Poor;<br>Poor;	

contentment during the past week?	Fair;<br>Good;<br>Very Good;	

Codes for Missing Data		
**Code / Value**	**Label**	**Example**
UNK	Unknown	Patient indicated that they did not know the answer. *Have you received the Hepatitis A vaccine?* If the patient indicates that they do not know the answer – then it should be marked as unknown.
NA	Not applicable	*If you checked off any problems, how difficult have these problems made it for you to do your work, take care of things at home, or get along with other people?* If the patient scored a 0 on the PHQ-9, then this question would be not applicable.
MISS	Missing	Incomplete medical records. *When did the patient start using benzodiazepines?* If there is no data entered, then the data is missing.
